# Dalbavancin in the Real-World Management of Gram-Positive Infections: A Systematic Review of Randomized and Observational Studies

**DOI:** 10.3390/microorganisms14051071

**Published:** 2026-05-09

**Authors:** Claudio Tana, Livia Moffa, Marco Tana, Samanta Moffa, Claudio Ucciferri

**Affiliations:** 1 Internal Medicine Unit, Eastern Hospital, ASL Taranto, 74024 Manduria, Italy; 2 Infectious Disease Clinic, G. D’Annunzio University of Chieti and ASL2 Lanciano Vasto Chieti, 66100 Chieti, Italy; 3 Internal Medicine Unit, G. D’Annunzio University of Chieti and ASL2 Lanciano Vasto Chieti, 66100 Chieti, Italy; 4 Department of Pharmacy, G. D’Annunzio University of Chieti, 66100 Chieti, Italy; 5 Infectious Disease Clinic, University of Perugia, 06123 Perugia, Italy

**Keywords:** dalbavancin, long-acting antibiotics, gram-positive infections, real-world evidence, outpatient parenteral antimicrobial therapy (OPAT)

## Abstract

Gram-positive infections are associated with significant morbidity and healthcare burden, often requiring prolonged intravenous therapy. Dalbavancin, a long-acting lipoglycopeptide, has emerged as a promising option beyond its approved indication for acute bacterial skin and skin structure infections (ABSSSI). A systematic review was conducted according to the PRISMA guidelines (PROSPERO: CRD420261296328). MEDLINE, Embase, CENTRAL, and Web of Science were searched from inception. Randomized controlled trials (RCTs) and observational studies evaluating dalbavancin in adult patients with Gram-positive infections were included. Outcomes of interest were clinical effectiveness, safety, and healthcare resource utilization. Risk of Bias was assessed using RoB 2 and the Newcastle–Ottawa Scale. Twenty-one studies were included. Randomized trials confirmed non-inferior efficacy of dalbavancin compared with standard therapy in ABSSSI. Observational studies demonstrated high clinical success rates across a range of infections, including osteo-articular infections, bloodstream infections, and infective endocarditis (IE), particularly in acute settings. Lower effectiveness was observed in biofilm-related infections without adequate source control. Dalbavancin was frequently used as sequential or consolidation therapy in complex patients. Its use was consistently associated with reduced length of hospital stay, facilitation of outpatient management, and potential cost savings. The safety profile was favorable, including in prolonged or multi-dose regimens. In conclusion, dalbavancin represents an effective and well-tolerated option for Gram-positive infections, with expanding evidence supporting its use in complex and off-label settings. Its pharmacokinetic profile enables simplified treatment strategies and improved healthcare resource utilization, although appropriate patient selection and source control remain essential.

## 1. Introduction

Gram-positive bacterial infections remain a major cause of morbidity and healthcare burden worldwide, encompassing a wide spectrum of clinical conditions ranging from acute bacterial skin and skin structure infections (ABSSSIs) to more complex and invasive diseases such as osteomyelitis, bloodstream infections, and infective endocarditis (IE) [1]. Their management is increasingly challenged by antimicrobial resistance, the need for prolonged intravenous therapy, and the growing clinical complexity of patients, including advanced age, multimorbidity, and frequent healthcare exposure [2].

Dalbavancin is a long-acting lipoglycopeptide antibiotic with potent activity against Gram-positive pathogens, including methicillin-resistant *Staphylococcus aureus* (MRSA), and has recently emerged as a promising therapeutic option [3]. Its unique pharmacokinetic profile, characterized by an extended half-life that allows for single- or two-dose regimens, offers relevant advantages in clinical practice, including improved adherence, reduced need for prolonged hospitalization, and facilitation of outpatient parenteral antimicrobial therapy (OPAT) [4]. Although originally approved for ABSSSIs, dalbavancin is increasingly used in real-world settings for a broad range of off-label indications, including bacteremia, bone and joint infections and IE [5].

Despite robust evidence from randomized controlled trials (RCTs) supporting its efficacy and safety in ABSSSI, the applicability of these findings to more complex infections and heterogeneous patient populations remains uncertain [6]. In parallel, an expanding body of observational and real-world evidence suggests that dalbavancin may be effective and well-tolerated across a wider spectrum of Gram-positive infections, with potential benefits in reducing hospital length of stay and overall healthcare resource utilization [7]. However, these data are still relatively limited and somewhat heterogeneous [8].

In this context, integrating evidence from both RCTs and real-world observational studies is essential to better define the clinical role of dalbavancin in contemporary practice. Bridging the gap between controlled trial settings and real-world clinical complexity is particularly relevant in patient populations that are often underrepresented in randomized studies, such as older adults with multiple morbidities [9].

Therefore, the aim of this systematic review is to comprehensively evaluate the clinical efficacy and safety of dalbavancin in the treatment of Gram-positive infections, synthesizing evidence from randomized and observational studies, and to explore its role across different infection types, clinical settings, and patient populations.

## 2. Materials and Methods

### 2.1. Study Design and Registration

This systematic review was conducted in accordance with the Preferred Reporting Items for Systematic Reviews and Meta-Analyses (PRISMA) guidelines. The protocol was prospectively registered in the PROSPERO database (registration number: CRD420261296328; registration date: 30 January 2026).

### 2.2. Search Strategy

A comprehensive systematic literature search was performed in the following electronic databases: MEDLINE (via PubMed), Embase, the Cochrane Central Register of Controlled Trials (CENTRAL), and Web of Science.

The search covered studies published from database inception to the most recent available date at the time of the search. No restrictions on publication status were applied. Only studies published in English were included.

The search strategy combined Medical Subject Headings (MeSH) and free-text terms related to dalbavancin, Gram-positive infections, acute bacterial skin and skin structure infections (ABSSSIs), osteomyelitis, bone and joint infections, bloodstream infections, IE, RCTs, observational studies, and real-world evidence.

In addition, the reference lists of included studies and relevant reviews were manually screened to identify further eligible studies.

### 2.3. Eligibility Criteria

Studies were selected according to the following PICO framework:

• Population: Adult patients (≥18 years) with confirmed or suspected Gram-positive infections.
• Intervention: Dalbavancin, administered according to any approved or off-label dosing regimen.
• Comparator: Standard intravenous antibiotics (e.g., vancomycin, daptomycin, or linezolid) or no comparator.
• Outcomes: Clinical effectiveness and safety outcomes.

Eligible study designs included RCTs, prospective and retrospective observational studies, cohort studies, case–control studies, and real-world registry studies.

Case reports, small case series (<10 patients), narrative reviews, editorials, and conference abstracts without full-text availability were excluded.

### 2.4. Study Selection and Data Extraction

Two reviewers (LM and SM) independently screened titles and abstracts for eligibility. Full texts of potentially relevant studies were subsequently assessed independently by the same reviewers. Disagreements were resolved by consensus or by consultation with a third reviewer (CT).

Data extraction included study design and setting, patient demographics, type of infection, dalbavancin dosing regimen, comparator treatments (when applicable), clinical outcomes, safety outcomes, and duration of follow-up.

### 2.5. Outcomes

The primary outcomes were clinical success (as defined by individual studies), clinical cure or improvement at the end of treatment, relapse or recurrence rates, and all-cause mortality.

Secondary outcomes included microbiological eradication, adverse events (overall and serious), treatment discontinuation due to adverse events, length of hospital stay, rehospitalization rates, outpatient parenteral antimicrobial therapy (OPAT) utilization, early discharge, and healthcare resource utilization, including cost-related outcomes when available.

### 2.6. Risk of Bias Assessment

The Risk of Bias was independently assessed by two reviewers (LM and SM). RCTs were evaluated using the Cochrane Risk of Bias tool (RoB 2), while observational studies were assessed using the Newcastle–Ottawa Scale (NOS). NOS scores were categorized as follows: 7–9 points indicate high quality, 5–6 moderate quality, and <5 low quality. For consistency across study designs, results are presented as categorized quality ratings in the corresponding table [10]. Discrepancies were resolved through discussion.

For non-comparative or single-arm studies, a descriptive adaptation of the assessment approach was applied, focusing on key methodological domains, including cohort selection, outcome definition and measurement, and adequacy of follow-up. This evaluation was used to support a critical interpretation of the methodological robustness of the evidence included in the narrative synthesis.

### 2.7. Data Synthesis

A qualitative narrative synthesis was performed, with results stratified by study design (randomized versus observational), type of infection, dosing regimen, and clinical setting (inpatient versus outpatient or OPAT).

## 3. Results

**Figure 1:**
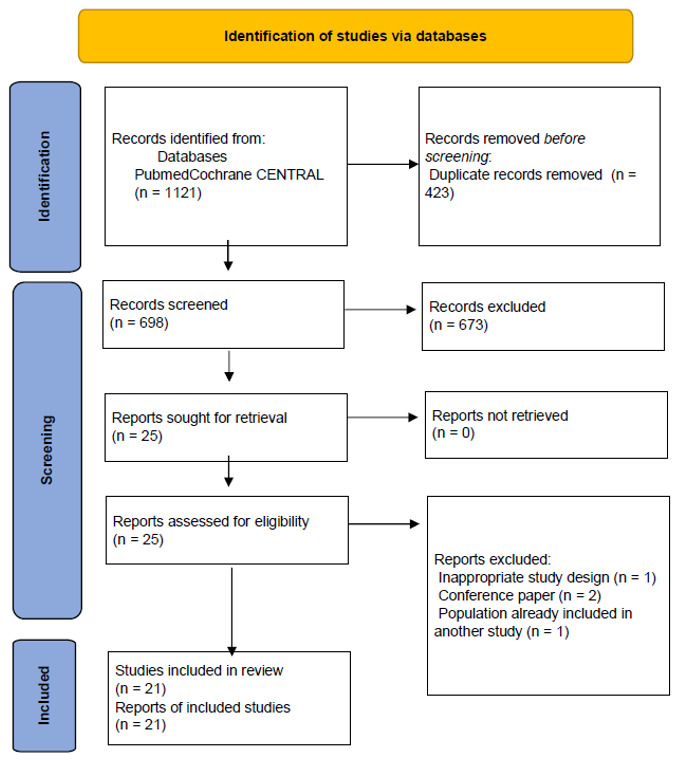
PRISMA flow diagram illustrating the study selection process, including identification, screening and final inclusion of studies.

The study selection process is summarized in Figure 1. A total of 1121 records were identified through database searching. After removal of 423 duplicates, 698 records were screened based on the title and abstract, of which 673 were excluded. Twenty-five full-text articles were assessed for eligibility, and four studies were excluded due to inappropriate study design (*n* = 1), conference abstract format (*n* = 2), or overlapping populations (*n* = 1). Overall, 21 studies were included in the final synthesis [11,12,13,14,15,16,17,18,19,20,21,22,23,24,25,26,27,28,29,30,31]. Table 1 summarizes the main characteristics of the studies included in this review, including study design, population, infection type, intervention details, and reported clinical outcomes.

### 3.1. Randomized Studies

In an open-label randomized clinical trial, Rappo et al. evaluated dalbavancin for the treatment of osteomyelitis in adults using a two-dose regimen (1500 mg on days 1 and 8) compared with standard-of-care antibiotic therapy administered for 4–6 weeks. Clinical cure at day 42 was achieved in 97% of patients receiving dalbavancin, compared with 88% in the standard therapy group. Notably, this benefit was sustained over time, with response rates of 96% maintained at both 6 months and 1 year. No treatment discontinuations due to adverse events were reported in the dalbavancin arm [11].

Beyond osteomyelitis, the most robust evidence in acute bacterial skin and skin structure infections (ABSSSIs) derives from the pivotal DISCOVER 1 and DISCOVER 2 trials. These phase 3, double-blind, non-inferiority studies randomized patients to receive intravenous dalbavancin on days 1 and 8 or intravenous vancomycin for at least 3 days, with an optional switch to oral linezolid to complete therapy [14].

The primary endpoint—early clinical response at 48–72 h—was similar between treatment groups (79.7% with dalbavancin vs. 79.8% with vancomycin/linezolid), demonstrating non-inferiority. Secondary endpoints at the end of treatment were also similar between groups. Among patients with *Staphylococcus aureus* infection, including methicillin-resistant strains, clinical success rates were high and comparable (90.6% with dalbavancin vs. 93.8% with comparator therapy). Importantly, dalbavancin was associated with a lower overall incidence of adverse events [14].

Table 2 presents the clinical effectiveness outcomes reported across the included studies, summarizing clinical cure rates, relapse or recurrence, and mortality, and illustrating the variability of results according to infection type and treatment strategy, while Table 3 presents the safety outcomes, including adverse events, serious adverse events, and treatment discontinuations, consistently indicating a favorable tolerability profile of dalbavancin across different clinical settings.

### 3.2. Skin and Soft Tissue Infections (ABSSSI)

RCTs have demonstrated that Dalbavancin is non-inferior to standard therapy (vancomycin followed, when appropriate, by oral linezolid) in the treatment of ABSSSI, with comparable clinical outcomes achieved through a simplified once-weekly dosing regimen.

In addition to randomized trials, real-world evidence consistently supports the role of dalbavancin in reducing hospitalization. In the ENHANCE study, McCarthy et al. evaluated the implementation of a dalbavancin-based clinical pathway in hospitalized patients with ABSSSIs. Compared with the pre-intervention period, dalbavancin use was associated with a significant reduction in infection-related length of stay (3.2 vs. 4.8 days), with similar clinical response rates (57% vs. 50%). Potential dalbavancin-related adverse events were reported in 17% of patients, while serious events were infrequent [16].

Consistent findings were reported in a multicenter retrospective study by Bai et al., including 228 patients with ABSSSIs treated in two Italian hospitals. Dalbavancin was associated with a significantly shorter length of stay (5.0 vs. 9.2 days) and comparable or slightly lower costs than the standard of care. Notably, first-line dalbavancin monotherapy was identified as the most favorable strategy in terms of both hospitalization duration and costs [17].

Additional real-world data were derived from a monocentric retrospective study conducted in the emergency department setting, where long-acting lipoglycopeptides were administered as single-dose therapy for SSTIs. In the cohort described by Ciusa et al., including patients treated with dalbavancin or oritavancin, 74% of patients were discharged without hospital admission. Clinical resolution at 14 days was achieved in 84% of cases, with a 10% recurrence rate at 30 days and no reported drug-related adverse events, rehospitalizations, or deaths. Although outcomes cannot be attributed exclusively to dalbavancin, these findings further support the potential role of long-acting lipoglycopeptides in facilitating emergency department management and reducing hospital admissions [31].

Overall, the available evidence indicates that, in ABSSSIs, dalbavancin achieves clinical outcomes comparable to standard therapy while contributing to reduced length of stay and simplification of care pathways.

### 3.3. Osteo-Articular Infections (Including Prosthetic Joint Infection)

For osteo-articular infections, comparative observational data further support the use of Dalbavancin. In a study by Simon et al., dalbavancin showed infection eradication rates comparable to standard therapy in prosthetic joint infections (77.5% vs. 74.2%), with similar rates of re-intervention (14.6% vs. 13.5%) [12].

Real-world evidence from Tobudic et al. (*n* = 72) reported an overall clinical cure rate of 64%, with better outcomes in acute infections (e.g., osteomyelitis, septic arthritis, and spondylodiscitis), while results were less favorable in biofilm-associated conditions, where adequate source control was a key determinant of success [13].

Consistent findings emerged from the OPAT experience reported by McSorley et al. (*n* = 102), with an overall cure rate of 67%. Outcomes were more favorable in acute conditions such as ABSSSIs (93%), bacteremia (100%), and acute osteomyelitis (90%), whereas lower success rates were observed in chronic osteo-articular and prosthetic infections, often managed with suppressive intent and suboptimal source control [15].

Additional evidence in prosthetic joint infections comes from the bicentric study by Mairesse et al. (*n* = 56), where dalbavancin used empirically in combination therapy achieved a 91.5% cure rate at a 2-year follow-up, with limited adverse events [21].

Finally, in diabetic foot osteomyelitis, Navarro-Jiménez et al. (*n* = 23) reported an 87% cure rate at 90 days. Dalbavancin was mainly used as second-line therapy in prolonged weekly regimens, with good tolerability [27].

### 3.4. Real-World Effectiveness Across Infection Types

Multicenter real-world evidence further supports the effectiveness of Dalbavancin across both on-label and off-label indications. In the Italian DALBITA study, which included 206 patients with skin and extra-skin infections, the overall clinical cure rate was 82.5%, with comparable effectiveness between ABSSSIs and off-label infections (85.5% vs. 75%), without significant differences [18].

Additional real-world data from a study by Parruti et al. (*n* = 100) included patients with ABSSSIs, bone and prosthetic infections, and cardiovascular infections. Dalbavancin was frequently administered in multi-dose regimens (mean of five infusions per patient), reflecting its use in complex infections. Clinical success rates were high both in on-label (84–91%) and off-label indications (82%). In multivariate analysis, higher loading doses were the only factor independently associated with improved outcomes. The safety profile was favorable, with no reported nephrotoxicity, neutropenia, or thrombocytopenia, and only two cases of self-limiting rash [19].

Consistent findings were reported by Arrieta-Loitegui et al. (*n* = 102), where approximately 70% of cases were off-label, mainly catheter-related bloodstream infections and endocarditis. All patients had received prior antibiotic therapy, and the main reason for switching to dalbavancin was to facilitate hospital discharge. Clinical and microbiological resolution was achieved in 93.7% of patients, with no infection-related readmissions within three months. Dalbavancin use was associated with a reduction of 14 hospital days per patient and an estimated cost saving of approximately €4550 per patient. Only one infusion-related allergic reaction was reported [22].

Finally, in the multicenter DALBADIA study, 97 diabetic patients with Gram-positive infections were treated with dalbavancin. The most frequent indications included cellulitis, prosthetic joint infection, endocarditis, and primary bacteremia. Simplification of antibiotic therapy was the main reason for its use. Among evaluable patients, 91.9% were classified as cured or clinically improved [29].

### 3.5. Bloodstream Infections and IE

Among the most relevant multicenter observational studies, Rebold et al. evaluated 115 patients receiving Dalbavancin as sequential therapy for Gram-positive bloodstream infections, including cases of IE. Conducted across 13 U.S. centers, the cohort included a clinically complex population, with a high proportion of people who inject drugs (40%) and frequent barriers to prolonged intravenous therapy. Dalbavancin was administered after a median of 10 days from index blood cultures, most commonly as a single 1500 mg dose. Clinical failure at 90 days occurred in 12.2% of patients, with a 90-day mortality of 7.0% and recurrence rate of 3.5%, supporting its role as a sequential option in selected patients where discharge is challenging [20].

Consistent findings were reported in a Spanish cohort by Hidalgo-Tenorio et al., including 83 hospitalized patients with bacteremia and/or IE. Clinical cure was achieved in 100% of bacteremia cases and 96.7% of endocarditis cases, with low failure and recurrence rates. Notably, dalbavancin use was associated with substantial reductions in hospital stay (over 1100 total days saved) and significant cost savings, without treatment discontinuation due to adverse events [23].

In elderly and comorbid patients, Aparicio-Minguijón et al. reported outcomes from 61 episodes of Gram-positive IE treated with dalbavancin as sequential therapy after a median of 27 days of standard antibiotics. The clinical cure rate at 6 months was 86.9%, with low relapse (1.6%) and limited treatment-related toxicity, alongside a marked reduction in hospitalization [24].

Further evidence in enterococcal endocarditis was provided in a multicenter cohort of 98 patients, where clinical cure was achieved in 81.2% at ≥12 months, with low recurrence (8.2%) and endocarditis-related mortality (3.1%). Surgical management was associated with improved outcomes, highlighting the importance of source control [25].

The EN-DALBACEN 2.0 cohort (*n* = 124) confirmed high effectiveness in complex endocarditis patients, with a 12-month cure rate of 95.9%, low recurrence (3.2%), and minimal adverse events. Dalbavancin was primarily used to facilitate early discharge, resulting in reduced hospital stay and lower healthcare costs [26].

Similarly, Brandariz-Núñez et al. described 48 elderly, comorbid patients treated with dalbavancin as consolidation therapy. While short-term success was high (93.8%), outcomes at 6 months were lower (77%), reflecting the impact of baseline frailty and comorbidity. Adverse events remained uncommon [28].

Finally, the large Italian multicenter SUSANA study (*n* = 281) provided a comprehensive real-world overview, including both on-label and off-label indications. Therapeutic success was comparable between the two groups (82.7% vs. 84.0%), with a favorable safety profile and very few serious adverse events [30].

Table 4 summarizes healthcare utilization outcomes, including length of hospital stay, readmission rates, OPAT use, and cost-related findings, highlighting the potential of dalbavancin to facilitate early discharge, reduce hospitalization, and optimize resource utilization.

### 3.6. Study Quality

The results of the Risk of Bias assessment are summarized in Table 5. RCTs showed an overall low Risk of Bias, with only minor concerns in selected domains. In contrast, observational studies were generally of moderate methodological quality, with higher-quality scores observed in multicenter and comparative designs.

Of the 21 included studies, 2 (9.5%) were RCTs and 19 (90.5%) were observational studies. Among the latter, 6 studies (31.6%) were classified as moderate-to-high or high quality, 11 (57.9%) as moderate quality, and 2 (10.5%) as moderate-to-low quality. Studies with higher methodological quality were typically multicenter cohorts and comparative designs.

## 4. Discussion

Dalbavancin has emerged as a valuable therapeutic option across a broad spectrum of Gram-positive infections, with growing evidence supporting its role beyond approved indications. Our findings are broadly consistent with those reported by Salvatore et al., who demonstrated comparable effectiveness and safety of dalbavancin versus standard of care in Gram-positive infections [3]. However, the present review extends these observations by incorporating a wider range of real-world studies and off-label indications, providing additional insight into its use in more complex and heterogeneous clinical scenarios.

Available data suggest that its efficacy is more pronounced in acute infections, whereas outcomes appear less favorable in biofilm-related settings when adequate source control is not achieved, highlighting the critical importance of combined medical–surgical management strategies [13]. This consideration is particularly relevant in outpatient contexts, including OPAT, where patient selection and infection characteristics are key determinants of success [15].

Dalbavancin consistently demonstrates a favorable safety and tolerability profile, even when administered in prolonged or multi-dose regimens. In complex, deep-seated, and off-label infections, real-world practice frequently adopts extended dosing strategies beyond the standard one- or two-dose schedules, with encouraging clinical outcomes [19]. These approaches reflect the need to tailor therapy to infection severity and site, particularly in cases such as osteo-articular infections or endovascular involvement. Overall, these findings suggest that dalbavancin represents a viable and effective option in osteo-articular infections, particularly in acute settings, with the additional advantage of simplified dosing and potential for outpatient management, although outcomes remain strongly dependent on adequate source control in chronic and biofilm-related infections. These considerations are consistent with real-world evidence highlighting the impact of infection severity, host factors, and timely therapeutic optimization on clinical outcomes, as also observed in studies of multidrug-resistant infections, where delayed or suboptimal management and higher inflammatory burden are associated with worse prognosis [32].

In bloodstream infections caused by Gram-positive organisms, dalbavancin has been increasingly utilized as a sequential or consolidation strategy in carefully selected patients, particularly when standard outpatient intravenous therapy is challenging. This is especially relevant for populations such as people who inject drugs, in whom maintaining long-term venous access may be problematic, or in frail elderly patients with multiple comorbidities, where minimizing hospitalization is a priority [11,24].

However, in these settings, outcomes must be interpreted cautiously, as they are influenced by baseline clinical complexity and typically follow an initial phase of conventional antibiotic therapy. Indeed, long-term outcomes in older, multimorbid patients may be limited by underlying frailty rather than infection control alone [28]. Overall, these findings support the role of dalbavancin as an effective sequential or consolidation strategy in bloodstream infections and IE, particularly in patients with complex clinical or logistical needs, while emphasizing the importance of appropriate patient selection and source control. Direct comparisons with standard therapies such as vancomycin, linezolid, and daptomycin are mainly available in ABSSSIs, where dalbavancin has shown non-inferiority [14]. In more complex or off-label infections, evidence is largely based on real-world studies without comparator groups, where dalbavancin is often used as sequential therapy after initial treatment with conventional antibiotics [20,23].

From a healthcare system perspective, dalbavancin enables early hospital discharge and facilitates outpatient management, translating into reduced hospital length of stay and potential cost savings without compromising clinical resolution [22]. Similar advantages have been observed in bloodstream infections, where its use as consolidation therapy in clinically stable patients supports both effectiveness and reduced hospitalization burden [23].

Particularly promising applications include difficult-to-treat infections such as IE, especially those caused by *Enterococcus* spp., which remain therapeutically challenging. Nevertheless, optimal management still requires adequate source control, often through surgical intervention combined with antimicrobial therapy [25]. Likewise, in osteomyelitis—including diabetic foot infections—dalbavancin represents a potential alternative, particularly in patients who are unable to tolerate prolonged conventional regimens or who develop treatment-related toxicity [27].

Recent real-world evidence indicates that the use of dalbavancin is progressively expanding beyond labeled indications, with comparable effectiveness and safety observed in both on-label and off-label settings [30]. In acute care settings such as emergency departments, long-acting agents like dalbavancin may contribute to reducing hospital admissions for selected skin and soft tissue infections, supporting more efficient patient flow and resource utilization [31].

However, these findings should be interpreted with caution. Studies evaluating multiple long-acting agents (e.g., dalbavancin and oritavancin) make it difficult to isolate the specific contribution of dalbavancin. In addition, most data on its off-label use derive from observational studies, predominantly of moderate methodological quality and subject to potential bias, including retrospective design, selection bias, confounding, and the absence of comparator groups. These limitations are particularly relevant in complex infections such as infective endocarditis and bloodstream infections, where dalbavancin is often used as sequential therapy following prior antibiotic exposure. Although the RCTs included in this review showed a generally low Risk of Bias, their evidence is largely limited to on-label indications. The lack of robust RCTs in off-label settings therefore limits the strength of current conclusions. Well-designed RCTs are needed to better define the efficacy, safety, and optimal use of dalbavancin in these contexts [31].

Furthermore, the overall heterogeneity across included studies in terms of study design, patient populations, infection types, and outcome definitions limited the comparability of the evidence and precluded the feasibility of conducting a quantitative meta-analysis and a formal assessment of publication bias. In addition, the variability and, in some cases, incomplete reporting of key variables did not allow for formal subgroup and sensitivity analyses.

Another limitation to consider is the potential risk of publication bias, as most included observational studies reported favorable outcomes, which may lead to an overestimation of dalbavancin effectiveness. Similarly, the potential risk of antimicrobial resistance with prolonged or widespread use should also be acknowledged. Current evidence on resistance development remains limited, particularly in real-world and off-label settings, and ongoing surveillance and prospective studies are needed to monitor resistance patterns and ensure the sustainable use of this agent [32]. Finally, although multi-dose regimens have been increasingly adopted in complex infections, the role of therapeutic drug monitoring (TDM) remains insufficiently explored. Whether TDM may be required to optimize efficacy and safety in severe infections, such as endocarditis, is still an open question and warrants further investigation [5].

Despite these constraints, such studies provide interesting insights into real-world practice, particularly regarding off-label use, extended dosing strategies, and outpatient management pathways, thereby complementing evidence derived from RCTs. Compared with standard therapies such as vancomycin, dalbavancin may offer several potential advantages in the management of resistant Gram-positive infections, including a prolonged half-life allowing for simplified dosing, reduced need for therapeutic drug monitoring, and a favorable safety profile. These features may translate into improved treatment adherence, lower risk of complications related to intravenous therapy, and reduced healthcare resource utilization, particularly in complex or outpatient settings [5,6].

Furthermore, the integration of artificial intelligence (AI) into clinical practice may further support the optimal use of Dalbavancin, particularly in complex patient populations [33]. AI-driven approaches may, in the future, help identify patients most likely to benefit from simplified long-acting regimens, particularly among older, multimorbid individuals in whom frailty, comorbidity burden, and infection complexity significantly influence therapeutic outcomes. By integrating clinical variables, microbiological data, and treatment history, AI models may support early prediction of treatment response and risk of failure, enabling more personalized therapeutic strategies. In addition, such tools could assist in optimizing dosing regimens and treatment duration, especially in complex or off-label scenarios, ultimately improving both efficacy and safety while enhancing resource utilization [34].

## 5. Conclusions

Dalbavancin is an effective and well-tolerated therapeutic option for Gram-positive infections, with increasing evidence supporting its use beyond approved indications. Its pharmacokinetic profile and simplified dosing make it particularly suitable for outpatient management, enabling early discharge and optimizing healthcare resource utilization.

Real-world data support its use in complex and off-label scenarios, including for deep-seated infections and use as sequential therapy, although clinical success remains closely dependent on appropriate patient selection and adequate source control.

Further high-quality studies are warranted to better define optimal treatment strategies and strengthen the evidence base for its broader clinical use.

## Figures and Tables

**Table 1:** Characteristics of included studies.

First Author, Year	Country	Study Design	Setting	Infection Type	Sample Size	Main Pathogen(s)	Dalbavancin Regimen	Comparator	Follow-Up
Rappo et al., 2019 [11]	Multicenter clinical trial/Ukraine	Randomized, open-label, comparator-controlled trial	Hospital-based	Osteomyelitis	Adults with first episode of osteomyelitis; *n* = 80 (DAL 70, SoC 10)	*Staphylococcus aureus* most common (60%)	1500 mg IV on day 1 and day 8	Standard of care (oral or IV antibiotics for 4–6 weeks)	Day 42, 6 months, 1 year
Simon et al., 2022 [12]	Multicenter database/Austria/Sweden	Retrospective propensity score-matched cohort	Orthopedic tertiary care centers	Periprosthetic joint infection (hip and knee)	*n* = 178 (DAL 89, SoC 89)	*Staphylococcus epidermidis*, *S. aureus*, *Cutibacterium* spp.	≥ 2 doses dalbavancin (variable regimen)	Standard of care antibiotics	≥ 1 year
Tobdic et al., 2019 [13]	Austria	Retrospective observational case series	Single tertiary care center	SSTI, osteomyelitis, spondylodiscitis, septic arthritis, PJI	*n* = 72	*S. aureus*, *S. epidermidis*	Variable dalbavancin regimens	None	End of dalbavancin therapy
Boucher et al., 2014 [14]	Multinational	Phase 3 double-blind non-inferiority RCT (pooled DISCOVER 1 and 2)	Multicenter trial	ABSSSI	*n* = 1312 (DAL 659, SoC 653)	*S. aureus and MRSA*	IV dalbavancin on days 1 and 8	IV vancomycin for ≥ 3 days with optional switch to oral linezolid	48–72 h and end of therapy
James C McSorley et al., 2024 [15]	UK	Retrospective observational cohort	OPAT service/hospital-based real-world cohort	ABSSSI, bacteraemia, acute/chronic osteomyelitis, native joint septic arthritis, PJI	*n* = 102	*Staphylococcus aureus*, *epidermidis*	IV dalbavancin on days 1 and 8	None	End of treatment
McCarthy et al., 2020 [16]	USA	Single-center pre–post comparative study	Urban tertiary care hospital	ABSSSI	*n* = 91 (pre 48, post 43)	*Staphylococcus aureus*	Dalbavancin pathway in post-period	Usual care in pre-period	44 days
Bai et al., 2023 [17]	Italy	Multicenter retrospective comparative study	2 hospital-based centers	ABSSSI	*n* = 228 (DAL 102; SoC 126)	*Staphylococcus aureus*, *S. epidermidis*	Dalbavancin, first/second/later line; monotherapy or combination	Standard of care antibiotics	During treatment
Bai et al., 2020 [18]	Italy	Multicenter retrospective observational study	11 hospital centers	ABSSSI (60.2%); other site infections (39.8%)	*n* = 206	*Staphylococcus aureus*, *S. epidermidis*	≥ 1 dose dalbavancin; often second-line or combination therapy	None	End of treatment
Parruti et al., 2024 [19]	Italy	Retrospective monocentric case series	Single-center real-world hospital cohort	ABSSSI (22%), bone/prosthetic infections (57%), cardiovascular infections (19%)	*n* = 100	MSSA 30%, MRSA 5%, MR-CoNS 20%, *Streptococcus* spp. 8%, no isolate in 32 cases	Multiple dose dalbavancin regimes; mean of 5 infusions	None	6 months
Rebold et al., 2024 [20]	USA	Multicenter retrospective observational cohort	13-center hospital-based real-world study	Gram-positive bloodstream infection, including IE	*n* = 115	*Staphylococcus aureus* (72%), coagulase-negative staphylococci (18%), *Streptococcus* spp. (16%)	Sequential dalbavancin therapy; most commonly single dose of 1500 mg	None	90 days
Mairesse et al., 2025 [21]	France	Retrospective bicentric observational study	2-center hospital-based cohort	Hip and knee prosthetic joint infection	*n* = 56	Gram-positive cocci predominated; all Gram-positive isolates susceptible to dalbavancin	Dalbavancin in combination with piperacillin-tazobactam as empirical intra-operative treatment	None	2 years
Arrieta-Loitegui et al., 2022 [22]	Spain	Retrospective observational single-center study	Tertiary hospital	Mixed Gram positive infections, off-label (catheter-related bacteremia and endocarditis)	*n* = 102	*Staphylococcus aureus*, *coagulase-negative staphylococci*, *Streptococcus* spp.	Dalbavancin after prior antibiotic therapy	None	End of treatment and 3 months for infection-related readmission
Hidalgo-Tenorio et al., 2019 [23]	Spain	Multicenter retrospective observational cohort	Hospital-based multicenter cohort	Bloodstream infection and IE due to Gram-positive cocci	*n* = 83	*Staphylococcus aureus* in BSI; coagulase-negative staphylococci in IE	Dalbavancin as consolidation therapy; at least 1 dose, regimen per clinical practice	None	In-hospital, 3 months, 1 year
Aparicio-Minguijón et al., 2025 [24]	Spain	Retrospective single-center observational cohort	Hospital-based real-world cohort	Definite IE due to Gram-positive bacteria	*n* = 61	*Staphylococcus aureus* (26.3%), *Enterococcus faecalis* (21.3%)	Sequential dalbavancin therapy; most commonly 1500 mg every 14 days	None	6 months
Hidalgo-Tenorio et al., 2025 [25]	Spain/France	Retrospective multicenter observational cohort	Multicenter hospital-based study	IE due to *Enterococcus* spp.	*n* = 98	*Enterococcus faecalis* (86.7%), *E. faecium* (11.2%)	Dalbavancin consolidation therapy; median total dose of 2500 mg over 3.5 weeks	None	≥12 months
Hidalgo-Tenorio et al., 2023 [26]	Spain	Retrospective multicenter observational cohort	Multicenter hospital-based study	IE due to Gram-positive cocci	*n* = 124	CoNS (38.8%), *Staphylococcus aureus* (22.6%), *Enterococcus faecalis* (19.4%), *Streptococcus* spp. (9.7%)	Dalbavancin sequential/consolidation therapy	None	≥12 months
Navarro-Jiménez et al., 2022 [27]	Spain	Retrospective descriptive single-center study	Multidisciplinary diabetic foot unit, second-level hospital	Diabetic foot infection with osteomyelitis	*n* = 23	*Staphylococcus aureus*, *Corynebacterium striatum*	Prolonged weekly dalbavancin regimens; commonly 1000 mg then 500 mg weekly	None	90 days after treatment
Brandariz-Núñez et al., 2024 [28]	Spain	Retrospective single-center observational cohort	Hospital-based real-world cohort	IE	*n* = 48	*Staphylococcus aureus* (45.8%), *Enterococcus* spp. (31.3%)	Dalbavancin as consolidation therapy	None	End of treatment and 6 months
Morata Ruiz et al., 2024 (DALBADIA) [29]	Italy/Spain	Retrospective multicenter observational cohort	Multicenter real-world cohort	Mixed Gram-positive infections in diabetic patients (cellulitis, PJI, endocarditis, bacteraemia)	*n* = 97	*Staphylococcus aureus*, *Staphylococcus epidermidis*, *Enterococcus faecalis*	Dalbavancin per clinical practice; one infusion in 34.8%, two infusions in 42.4%	None	End of observation
Tordi et al., 2025 (SUSANA cohort) [30]	Italy	Retrospective multicenter observational cohort	Multicenter real-world surveillance cohort	Mixed Gram-positive infections (on-label and off-label)	*n* = 281	MRSA prominent in off-label targeted therapy; mixed pathogens	Dalbavancin per routine practice; on-label and off-label regimens	None	Not clearly specified in abstract
Ciusa et al., 2025 [31]	Italy	Retrospective observational cohort	Emergency room, tertiary care hospital	SSTIs	*n* = 19	*Staphylococcus aureus*, *Staphylococcus epidermidis*, *Enterococcus faecalis*	Single-dose dalbavancin 1500 mg or oritavancin 1200 mg	None	14 and 30 days

**Table 2:** Clinical effectiveness outcomes.

Study	Infection Type	Primary Effectiveness Outcome	Clinical Success/Cure	Relapse/Recurrence	Mortality	Main Findings
Rappo et al., 2019 [11]	Osteomyelitis	Clinical response at day 42	97% (65/67) DAL vs. 88% (7/8) SoC	Sustained response in DAL group at 6 months and 1 year (96%)	Not major outcome	Dalbavancin showed high cure rates and durable response
Simon et al., 2022 [12]	Periprosthetic joint infection (hip and knee)	Infection eradication/re-revision rates	77.5% DAL vs. 74.2% SoC	Re-revision 14.6% DAL vs. 13.5% SoC	Not major outcome	Dalbavancin showed similar effectiveness to standard of care in PJI
Tobudic et al., 2019 [13]	Mixed Gram-positive infections	Clinical cure at the end of dalbavancin therapy	64% achieved cure without additional antibiotics	Not reported	Not reported	Dalbavancin appeared most effective in acute SSTI, acute osteomyelitis, septic arthritis and spondylodiscitis; source control was critical in biofilm-associated infections
Boucher et al., 2014 [14]	ABSSSIs	Early clinical response at 48–72 h	79.7% DAL vs. 79.8% vancomycin-linezolid; non-inferior	Not reported	Not reported	Dalbavancin was non-inferior to vancomycin-linezolid for ABSSSI
James C McSorley et al., 2024 [15]	Mixed Gram-positive infections	Cure at end of treatment	Overall cure achieved in 67%; ABSSSIs in 93%; bacteraemia in 100%; acute osteomyelitis in 90%; native joint septic arthritis in 75%; PJI in 33%	Suppressive success: chronic osteomyelitis 48% PJI 66%	Not reported as main outcome	Dalbavancin was effective in ABSSSI; poor source control was associated with worse outcomes in chronic bone/joint infections
McCarthy et al., 2019 [16]	ABSSSIs	Complete response during follow-up	57% post-period vs. 50% pre-period	Not reported	Not reported as main outcome	Dalbavancin pathway achieved similar clinical response while reducing hospital stay
Bai et al., 2023 [17]	ABSSSIs	Comparative effectiveness in real-world practice	Dalbavancin associated with favorable effectiveness as first-line monotherapy	Not reported	Not reported	First-line dalbavancin monotherapy appeared efficacy for ABSSSI management
Bai et al., 2020 [18]	ABSSSIs and other site infections	Clinical cure at end of treatment	Overall, 82% achieved clinical cure; ABSSSI 85.5% vs. OTA 75% (NS)	Not reported	Not reported	High effectiveness across indications; similar efficacy in ABSSSIs and off-label infections
Parruti et al., 2024 [19]	Mixed Gram-positive infections	Clinical success at follow-up	84% for registered indications; unregistered indications	Not reported	Not reported	Dalbavancin showed favorable effectiveness in label and off label infections; higher loading doses were associated with better outcomes
Rebold et al., 2024 [20]	Gram-positive bloodstream infection	Composite clinical failure at 90 days	Composite clinical failure in 12.2%	90-day BSI recurrence 3.5%	90-day mortality 7.0%	Dalbavancin appeared useful as sequential therapy in Gram-positive BSI, particularly to facilitate hospital discharge
Mairesse et al., 2025 [21]	Prosthetic joint infection	Absence of relapse during 2-year follow-up	Cure rate 91.5%	Treatment failure in 4 patients	Not reported as main outcome	Empirical dalbavancin-based combination therapy was associated with high 2-year cure rates in PJI
Arrieta-Loitegui et al., 2022 [22]	Mixed Gram-positive infections	Clinical and microbiological resolution plus no admission due to same infection within 3 months	93.7% clinical and microbiological resolution	No infection-related admission within 3 months included in effectiveness definition	Not emphasized	Dalbavancin was effective in routine practice, particularly as an off-label discharge strategy
Hidalgo-Tenorio et al., 2019 [23]	BSI and IE	Clinical response during hospitalization, at 3 months and 1 year	IE effectiveness 96.7%; BSI clinical cure 100% during hospitalization and at 3 months	IE therapeutic failure in 2.9%; no BSI recurrence reported	IE: 8.8% mortality unrelated to IE; no BSI deaths reported	Dalbavancin was effective as consolidation therapy in clinically stabilized patients with IE and BSI
Aparicio-Minguijón et al., 2025 [24]	IE	Clinical cure at 6 months	86.9% clinically cured	1.6% relapse	11.5% 6-month mortality; 1.6% IE-related death	Dalbavancin showed high effectiveness as sequential therapy in elderly and comorbid IE patients
Hidalgo-Tenorio et al., 2025 [25]	Enterococcal IE	Clinical cure at ≥12 months	81.2% clinically cured	8.2% relapse	3.1% 1-year IE-related mortality	Dalbavancin appeared effective as consolidation therapy for enterococcal IE, particularly when combined with adequate source control/surgery
Hidalgo-Tenorio et al., 2023 [26]	IE	Effectiveness at 12 months	95.9% effectiveness	3.2% relapse	0.8% IE-related death	Dalbavancin was highly effective as sequential/consolidation therapy in Gram-positive IE
Navarro-Jiménez et al., 2022 [27]	Diabetic foot infection/osteomyelitis	Cure at 90 days after treatment completion	87% (20/23)	Not reported	Not reported	Dalbavancin showed high cure as part of multidisciplinary treatment for diabetic foot osteomyelitis
Brandariz-Núñez et al., 2024 [28]	Infective endocarditis	Effectiveness at the end of treatment and 6 months	93.8% at the end of treatment; 77% at 6 months	2 relapses at 6 months	6 IE-related deaths and 4 unrelated deaths at 6 months	Dalbavancin was effective as consolidation therapy in elderly/comorbid IE patients, although effectiveness declined over follow-up due to relapse and mortality
Morata Ruiz et al., 2024 (DALBADIA) [29]	Mixed Gram-positive infections in diabetic patients	Clinical cure or improvement at end of observation	91.9% clinically cured	Not reported	Not reported	Dalbavancin showed high rates of positive clinical response in diabetic patients across multiple infection types
Tordi et al., 2025 (SUSANA cohort) [30]	Mixed Gram-positive infections	Clinical cure or infection control	82.7% on-label; 84% off-label	Not reported	Not reported	Dalbavancin showed comparable effectiveness in both approved and off-label indications
Ciusa et al., 2025 [31]	SSTIs	Clinical resolution at day 14	84% achieved clinical resolution at 14 days	10% recurrence at 30 days	Not reported	Long-acting lipoglycopeptides appeared effective in ER-managed SSTIs and support early discharge

**Table 3:** Safety outcomes.

Study	Adverse Events	Serious Adverse Events	Discontinuation Due to AE	Specific Safety Notes	Safety Conclusion
Rappo et al., 2019 [11]	AEs reported in 10 dalbavancin-treated patients	Not emphasized as major issue	None	Dalbavancin was well tolerated	Favorable safety profile
Simon et al., 2022 [12]	Low rate of adverse events	Not detailed	None	Dalbavancin was well tolerated	Favorable safety profile
Tobudic et al., 2019 [13]	4/72 (5%) AEs	Not emphasized	Not reported	Nausea, rash/exanthema, hyperglycemia	Favorable safety profile
Boucher et al., 2014 [14]	AEs less frequent with dalbavancin	Not reported	Not reported	Nausea, diarrhea, pruritus	Favorable safety profile compared with vancomycin-linezolid
James C McSorley et al., 2024 [15]	14/102 AEs	Not reported	Not reported	Real-world tolerability acceptable	Dalbavancin was well tolerated
McCarthy et al., 2019 [16]	Possible dalbavancin-related AEs in 17% (7 patients)	Serious AEs: 7% post-period vs. 2% pre-period	Non reported	Few serious AEs overall	Acceptable safety
Bai et al., 2020 [18]	11/206 (5.4%) non-serious AEs	Not reported	Not reported	No major safety concerns reported	Favorable safety profile in real-world use
Parruti et al., 2024 [19]	2 mild skin rashes	Not reported	Not reported	No renal toxicity, neutropenia or thrombocytopenia observed during treatment or follow-up	Favorable safety profile in prolonged multi dose use
Mairesse et al., 2025 [21]	Few adverse events, mainly digestive (diarrhea, pain)	Not clearly reported	Not reported	Tolerability appeared favorable	Dalbavancin-based empirical therapy was generally well tolerated
Arrieta-Loitegui et al., 2022 [22]	One allergic reaction during infusion	Not otherwise	1 patient did not complete infusion	Overall good tolerability	Favorable safety profile in real-world off-label use
Aparicio-Minguijón et al., 2025 [24]	AEs in 8.2% of patients	Only one event (1.6%) attributed to dalbavancin (infusion reaction)	Not clearly reported as discontinuation	Overall excellent tolerability	Favorable safety profile in elderly/comorbid IE patients
Hidalgo-Tenorio et al., 2025 [25]	Minimal adverse events reported	Severe AEs in 1% (acute tubular necrosis)	Not clearly reported	Overall good tolerability	Favorable safety profile in enterococcal IE consolidation therapy
Navarro-Jiménez et al., 2022 [27]	Mild side effects in 3 patients (nausea/GI discomfort)	None reported	Not reported	Good tolerability during prolonged treatment	Favorable safety profile
Brandariz-Núñez et al., 2024 [28]	Dalbavancin related AEs in 4.2%	Serious AEs in 2%	Not clearly reported	Few treatment-related adverse effects	Favorable safety profile
Tordi et al., 2025 (SUSANA cohort) [30]	Few AEs overall	One grade-3 AE in each cohort	Only 1 AE led to discontinuation	Good tolerability in both on-label and off-label use	Favorable safety profile

**Table 4:** Healthcare resource utilization, OPAT, and cost outcomes.

Study	Length of Stay Impact	Readmission	Economic Findings	Resource-Use Conclusion
James C McSorley et al., 2024 [15]	OPAT use implied avoidance of prolonged inpatient treatment	Not reported	Not reported	Dalbavancin supported outpatient management of deep-seated infections
McCarthy et al., 2019 [16]	Mean infection-related LOS reduction from 4.8 to 3.2 days	Not reported	Not reported	Dalbavancin reduced hospital stay and improved work productivity impairment
Bai et al., 2023 [17]	LOS reduction with dalbavancin (5.0 vs. 9.2 days)	Not reported	Lower mean direct medical costs with dalbavancin	Dalbavancin as first-line monotherapy reduced LOS and supported cost-saving ABSSSI management
Bai et al., 2020 [18]	Longer LOS in OTA vs. ABSSSI (13.5 vs. 3 days)	Not reported	Not reported	Dalbavancin used across settings; more complex infections required longer hospitalization
Rebold et al., 2024 [20]	Dalbavancin used to facilitate hospital discharge after median 10 days from index culture	Composite failure includes healthcare reutilization at 90 days	Not reported	Sequential dalbavancin may support earlier discharge in patients requiring prolonged parenteral therapy
Arrieta-Loitegui et al., 2022 [22]	Median reduction in LOS of 14	No admission due to same infection within 3 months included in effectiveness outcome	Estimated saving of ~€4550 per patient	Dalbavancin facilitated early discharge and outpatient management
Hidalgo-Tenorio et al., 2019 [23]	Hospital stay reduction of 636 days for BSI and 557 days for IE	No BSI recurrence/readmission reported in follow-up	Estimated savings: €315,424.20 for BSI and €283,187.45 for IE	Dalbavancin consolidation therapy reduced hospital stay and appeared cost-effective
Aparicio-Minguijón et al., 2025 [24]	Total hospitalization reduction of 1090 days	Not specifically reported	Not directly reported	Dalbavancin allowed substantial reduction in in-hospital stay in sequential IE therapy
Hidalgo-Tenorio et al., 2025 [25]	Hospital stay reduction of 21 days (14–28)	Not specifically reported	Not directly reported	Dalbavancin facilitated discharge in 88.8% of patients and substantially reduced hospitalization
Brandariz-Núñez et al., 2024 [28]	Dalbavancin mainly used to facilitate OPAT (85.4%)	Not specifically reported	Not directly reported	Dalbavancin as consolidation therapy in comorbid IE patients
Ciusa et al., 2025 [31]	74% discharged without hospital admission	No readmission reported	Not reported	ER use of long-acting lipoglycopeptides supported early discharge and reduced hospitalization burden

**Table 5:** Risk of Bias and methodological quality assessment of included studies. Newcastle–Ottawa Scale (NOS) scores were calculated for all observational studies and categorized as follows: 7–9 points indicate high quality, 5–6 moderate quality, and <5 low quality. Randomized controlled trials were assessed using the Cochrane Risk of Bias 2 (RoB 2) tool and are reported accordingly.

Study	Selection Bias	Deviations from Intended Interventions	Missing Outcome Data	Outcome Measurement	Overall
Boucher et al., 2014 [14]	Low	Low	Low	Low	Low risk
Rappo et al., 2019 [11]	Some concerns	Some concerns	Low	Some concerns	Some concerns
Simon et al., 2022 [12]	Low	Low	Low	Low	Moderate–high quality
Bai et al., 2023 [17]	Some concerns	Some concerns	Low	Low	Moderate quality
McCarthy et al., 2020 [16]	Some concerns	Some concerns	Some concerns	Low	Moderate–low quality
Tobdic et al., 2019 [13]	Some concerns	Some concerns	Some concerns	Some concerns	Moderate quality
McSorley et al., 2024 [15]	Some concerns	Some concerns	Some concerns	Some concerns	Moderate quality
Parruti et al., 2024 [19]	Some concerns	Some concerns	Low	Low	Moderate quality
Arrieta-Loitegui et al., 2020 [22]	Some concerns	Some concerns	Low	Low	Moderate quality
Hidalgo-Tenorio et al., 2019 (DALBACEN) [23]	Low	Low	Low	Low	Moderate–high quality
Aparicio-Minguijón et al., 2024 [24]	Some concerns	Some concerns	Low	Low	Moderate quality
Hidalgo-Tenorio et al., 2025 [25]	Low	Low	Low	Low	Moderate–high quality
Hidalgo-Tenorio et al., 2023 (EN-DALBACEN 2.0) [26]	Low	Low	Low	Low	Moderate–high quality
Brandariz-Núñez et al., 2024 [28]	Some concerns	Some concerns	Some concerns	Low	Moderate quality
Navarro-Jiménez et al., 2022 [27]	Some concerns	Some concerns	Some concerns	Low	Moderate quality
Morata Ruiz et al., 2024 (DALBADIA) [29]	Some concerns	Some concerns	Some concerns	Low	Moderate quality
Tordi et al., 2025 (SUSANA) [30]	Low	Low	Low	Low	Moderate–high quality
Ciusa et al., 2025 [31]	Some concerns	Some concerns	Some concerns	Some concerns	Moderate–low quality
Mairesse et al., 2025 [21]	Some concerns	Some concerns	Low	Low	Moderate quality
Rebold et al., 2024 [20]	Low	Low	Low	Low	Moderate–high quality
Bai et al., 2020 (DALBITA) [18]	Some concerns	Some concerns	Some concerns	Low	Moderate quality

## Data Availability

No new data were created or analyzed in this study.
